# Discovery of New Microbial Collagenase Inhibitors

**DOI:** 10.3390/life12122114

**Published:** 2022-12-15

**Authors:** Georgiana Nitulescu, Dragos Paul Mihai, Anca Zanfirescu, Miruna Silvia Stan, Daniela Gradinaru, George Mihai Nitulescu

**Affiliations:** 1 Faculty of Pharmacy, “Carol Davila” University of Medicine and Pharmacy, Traian Vuia 6, 020956 Bucharest, Romania; 2 Department of Biochemistry and Molecular Biology, Faculty of Biology, University of Bucharest, 91-95 Spl. Independentei, 050095 Bucharest, Romania; 3 Research Institute of the University of Bucharest (ICUB), University of Bucharest, 050657 Bucharest, Romania

**Keywords:** capsaicin, curcumin, 4′,5-dihydroxyflavone, juglone, palmatine chloride, *Clostridium histolyticum*, antivirulence

## Abstract

**Simple Summary:**

Antimicrobial resistance is spreading globally, making healthcare specialists fear “a return to the dark age of medicine”. Great efforts are being made to develop new antimicrobials or to repurpose discontinued or shelved drugs to be used against resistant “superbugs”. Resistance occurs partly because the antibiotics we use kill the bacteria. However, this also induces a high selection pressure: a resistant bacterium will multiply, while its non-resistant competitors will die. Thus, resistance will develop rapidly in the presence of that antibiotic. An alternative is simply disarming bacteria—reducing their ability to infect the host. In other words, we should develop drugs that inhibit bacterial virulence rather than bacterial viability. Bacterial virulence factors such as collagenases emerged as attractive targets for overcoming antimicrobial resistance. Microbial collagenases digest host collagen, ensuring the spread of the pathogen and its toxins into host tissues. In this study, we aimed to identify plant-derived and repurposed drugs which potently inhibit collagenase A. We achieved this by performing extensive screening. We identified capsaicin, 4′,5-dihydroxyflavone, curcumin, dihydrorobinetin, palmatine chloride, biochanin A, 2′-hydroxychalcone, and juglone as promising collagenase inhibitors. Our data indicate these molecules could be used against infections caused by multi-drug-resistant bacterial pathogens which express collagenase A.

**Abstract:**

Bacterial virulence factors are mediating bacterial pathogenesis and infectivity. Collagenases are virulence factors secreted by several bacterial stains, such as *Clostridium*, *Bacillus*, *Vibrio* and *Pseudomonas*. These enzymes are among the most efficient degraders of collagen, playing a crucial role in host colonization. Thus, they are an important target for developing new anti-infective agents because of their pivotal roles in the infection process. A primary screening using a fluorescence resonance energy-transfer assay was used to experimentally evaluate the inhibitory activity of 77 compounds on collagenase A. Based on their inhibitory activity and chemical diversity, a small number of compounds was selected to determine the corresponding half maximal inhibitory con-centration (IC50). Additionally, we used molecular docking to get a better understanding of the enzyme–compound interaction. Several natural compounds (capsaicin, 4′,5-dihydroxyflavone, curcumin, dihydrorobinetin, palmatine chloride, biochanin A, 2′-hydroxychalcone, and juglone) were identified as promising candidates for further development into useful anti-infective agents against infections caused by multi-drug-resistant bacterial pathogens which include collagenase A in their enzymatic set.

## 1. Introduction

Owing to their high adaptability to environmental changes, bacteria quickly learn to escape the attack of antibiotics. This results in a decrease in the efficacy of antibiotics [1]. Exposure of bacteria to high concentrations or long courses of antibiotics promotes an increase in resistant mutants while counteracting the susceptible strains [2]. Over time, the over- or misuse of antibiotics led to a high number of bacterial populations resistant to multiple antimicrobials, with tragic impact on the morbidity and mortality associated with numerous infectious diseases [3,4]. One important underlying cause resides in the mechanism of the majority of the antibiotics: they either kill bacteria (bactericidal) or inhibit their growth (bacteriostatic), thus promoting the selection of resistant variants [5]. One strategy to circumvent this problem consists of disarming rather than killing the bacterial pathogens [6]. Various therapeutic alternatives to conventional antibiotics are being pursued, such as bacteriophages, antivirulence and/or antibiofilm agents, probiotics, vaccines, immune stimulation therapies, and antibodies [7]. The antivirulence agents, also known as anti-infectives, target the pathogens’ mechanisms of inducing disease without impacting their survival [8].

Promising antivirulence strategies include the inhibition of pore-forming toxins [9], pili biogenesis by targeting chaperone-usher pathway [10], attachment of surface proteins to the peptidoglycan wall of Gram-positive bacteria by targeting sortases [11], quorum-sensing regulatory proteins [12], as well as interference with the quorum-sensing signal detection [13], disruption of the biosynthesis of functional membrane microdomains [14], and reduction of biofilm formation or distortion of its structure [15].

Furthermore, the pathogenicity of many bacteria is associated with the production and dissemination of tissue-destructive enzymes that help the bacteria to invade the host organism. The bacterial collagenases are such proteolytic enzymes, cleaving both water- insoluble and -soluble collagens and subsequently promoting the destruction of some extracellular structures and the penetration into anaerobic sites, spreading the infection [16]. Their inhibition is a conceptually attractive antimicrobial strategy, as it should block the colonization and infiltration of the host by the bacteria, reducing the Darwinian selection pressure [17]. Furthermore, targeting extracellular collagenases would offer another advantage: inhibitors do not need to cross the bacterial cell wall, which is challenging in many cases [18]. 

The majority of mature collagenases comprise an N-terminal collagenolytic unit of approximately 78 kD and two or three accessory domains with various functions such as binding to fibrillar collagen [19]. The peptidase domain harbors a catalytic zinc coordinated by the two histidine residues and one glutamate [20]. 

Several groups developed clostridial collagenase inhibitors, focusing on collagenases G (ColG) and H (ColH) from *C. histolyticum*. This included sulfonylated derivatives of amino-acid hydroxamates [21,22], 5-amino-2-mercapto-1,3,4-thiadiazoles [23], aryl sulfonyl-urea derivatives [24], and N-aryl mercaptoacetamides [25]. However, the synthetic collagenase inhibitors were associated with a poor safety profile unsuitable for antibacterial therapy in humans. This may be explained, at least partially, by their mechanism of action: the zinc-binding group in their structure irreversibly chelates the catalytic zinc ion [26]. 

We posit that natural compounds would provide an attractive alternative to synthetic collagenase inhibitors, as some studies indicate that plant-derived oligosaccharides, polyphenols, and fatty acids bind reversibly to the catalytic zinc [27,28]. However, information on the topic is scarce. Another strategy which would allow identification of new collagenase inhibitors with an acceptable margin of safety is drug repurposing—these inhibitors would have well-known adverse reactions.

In this study, we aimed to identify plant-derived inhibitors of collagenase A, as well as synthetic compounds with a well-known safety profile, which could be further used as novel adjuvant antibacterial therapy. We performed an extensive initial screening evaluating the inhibitory activity of 77 compounds on collagenase A. 

For the most promising derivatives, we further determined the corresponding half maximal inhibitory concentration (IC50) and performed molecular modeling to understand the potential interactions between the isolated compounds and the enzyme.

## 2. Materials and Methods

### 2.1. Collagenase Activity Assay

The inhibitory activity of all compounds was determined by quantifying the increase in fluorescence intensity upon cleavage of the BODIPY substrate, a type B gelatin conjugate, using the AB211108 Collagenase Inhibitor Assay Kit (BioVision, San Francisco, USA). According to the kit protocol, reactions were performed in a 96-well plate, each well containing 1 µL test compound solution, 5 µL collagenase (ColA) solution, 44 µL buffer solution, and 50 µL collagenase substrate solution. All compounds were dissolved in dimethylsulfoxide (DMSO). The final concentration of DMSO in the sample was 1%. The sample composed of 45 µL buffer, 5 µL enzyme solution, and 50 µL collagenase substrate solution was considered the positive control. The samples containing 1 µL solution of 1,10-phenanthroline—the kit inhibitor, 5 µL enzyme solution, 44 µL buffer solution, and 50 µL collagenase substrate solution represented the negative controls (I). These were performed at 5 concentrations, namely 500 µM, 400 µM, 300 µM, 200 µM, and 100 µM. A sample containing 50 µL buffer and 50 µL substrate solution (S) was prepared in order to observe the substrate functionality. The fluorometric analysis was performed using the FlexStation 3 Multi-Mode Microplate Reader (Molecular Device, San Francisco, CA, USA), at Ex/Em = 485 nm/535 nm after a 15 min incubation period at 37 °C. Twelve recordings were made every 5 min starting at 0, considered after the 15 min of incubation. All reported values are the means of triplicate assays.

All reagents and solvents were purchased from commercial suppliers. The 77 compounds used for screening were purchased from Sigma-Aldrich (St. Louis, MO, USA) and were as follows: 1,2-diaminoanthraquinone; 1,2,3,4-tetrahydro-beta-carboline-1-carboxylic acid; acetylcysteine; acamprosate calcium; acetohexamide; aloin; apigenin; *L*-arginine; argatroban; artemisinin; bendroflumethiazide; benfotiamine; benzoyl-L-arginine ethyl ester hydrochloride; berberine; biochanin A; caffeic acid; cantharidin; capsaicin; captopril; chlorogenic acid; chrysophanol; citrulline; chrysin; curcumin; cysteamine hydrochloride; cysteine; daidzein; daidzein dimethyl ether; 1,4-dihydroxyanthraquinone; 3,6-dihydroxyflavone; 4′,5-dihydroxyflavone; 4,6-dihydroxy-2-mercaptopyrimidine; dihydrorobinetin; *meso*-2,3-dimercaptosuccinic acid; epicatechin; esculetin; etidronic acid; ferulic acid; Fmoc-*L*-citrulline; gallic acid; genistein; 2′-hydroxychalcone; 3-hydroxycinnamic acid; 6-hydroxycoumarin; 3-hydroxyflavone; isoxicam; isorhamnetin; juglone; lawsone; 4′-methoxyflavonol; 6-methoxyflavonol; 6-methoxy-1,2,3,4-tetrahydro-9H-pyrido [3,4-b]indole-1-carboxylic acid; myricetin; myricitrin; nimesulide; palmatine chloride; 3-phenyl-1*H*-2-benzopyran-1-one; piperine; plumbagin; podophyllotoxin; potassium clavulanate; primuletin; rhein; shikimic acid; tetrahydronorharman; trihydroxyethylrutin; umbelliferone; usnic acid; and from AlfaAesar: *L*-alanine; S-(acetamidomethyl)-*L*-cysteine; N-(3-chlorophenylsulfonyl)-*DL*-alanine; 4-methylphenylsulfonylurea; nalpha-*p*-toluenesulfonyl-*L*-arginine; sodium 2- mercaptoethanesulfonate; sulbactam; tazobactam; 1-{[3-(trifluoromethyl)phenyl]sulfonyl}-2-pyrrolidinecarboxylic acid.

All the compounds were first tested at a concentration of 100 μM, and based on the results of this preliminary screening, a series of 32 compounds were selected to be further tested at 5 concentrations (1 μM, 5 μM, 10 μM, 50 μM, and 100 μM) in order to evaluate their inhibitory activity.

### 2.2. Molecular Docking

A molecular docking experiment was carried out to investigate the binding energies of the screened compounds and the potential binding poses that could explain the biological activity. Since there is no readily available crystal structure of *C. perfringens* ColA, in our study, we used a validated homology model of the target protein that we reported in a previous paper [29]. The docking experiment was carried out using the AutoDock Vina v1.1.2 [30] algorithm within YASARA Structure [31]. The docking search space was established around the peptidase domain, the active site including the catalytic Zn^2+^, which is complexed by histidine residues 502 and 506, and histidine-stabilizing glutamate residues 503 and 534.

Protein and ligand structures were protonated according to the physiological pH (7.4). Conformations of the screened ligands were generated using DataWarrior 5.2.1 [32] and were minimized with MMFF94s+ forcefield. A total of 12 docking runs was performed for each compound and the results were retrieved as the binding energy (ΔG, kcal/mol) and ligand efficiency (ΔG\no. of heavy atoms). Molecular interactions and binding poses were analyzed using BIOVIA Discovery Studio Visualizer (BIOVIA, Discovery Studio Visualizer, Version 17.2.0, Dassault Systèmes, 2016, San Diego, CA, USA).

### 2.3. Statistical Analysis

Statistical analysis of the inhibitory effect on ColA was performed using GraphPad Prism version 5.01 software (GraphPad Software, Inc., La Jolla, CA, USA). The inhibition (I %) values were calculated and plotted against the logarithm of concentrations. The corresponding curves were calculated using the least squares fit method. Whenever the obtained results permitted, the upper and lower limits of the 95% confidence interval (95% CI) and the determination coefficient (*r^2^*) were calculated. Pearson’s correlation test was used to assess the potential correlation between experimental and predicted activity values.

## 3. Results

### 3.1. Collagenase Activity Assay

A total of 77 compounds were chosen for the screening on ColA activity based on their molecular diversity and on a set of rules derived from our previous research [29] based on 2D molecular descriptors and Bemis–Murcko scaffolds. The first group of compounds contained natural compounds, while the second one contains synthetic derivatives of several amino acids. The first group comprised 4 derivatives of cinnamic acid, 3 naphthoquinones, 5 anthraquinones, 3 coumarins, 20 members of the flavonoid family, 3 alkaloids, and 8 other compounds with heterogenous structures. The second group contained 12 derivatives of various amino acids and 19 chemically diverse compounds. The percentage inhibition values after incubation at 100 μM are presented in Table S1. 

The inhibitory activity was defined as the concentration of compound causing a 50% decrease in ColA activity (IC50), relative to the negative control after 30 min of exposure. A total of 32 compounds were chosen for determination of the IC50 based on their preliminary inhibition results and to provide good chemical diversity. The results of the five-dose assay are presented in Table 1 as average percentage inhibition values and their standard deviation (SD). We listed only the compounds which had a significant inhibitory effect in the range of concentrations used.

Almost complete inhibition of the enzyme activity was registered for capsaicin at a concentration of 1 µM, 4′,5-dihydroxyflavone and curcumin at concentrations of 5 µM, dihydrorobinetin at 10 µM, palmatine chloride and biochanin A at 50 µM, and 2′-hydroxychalcone and juglone at 100 µM. The IC50 values were calculated for only five compounds due to the fact that for other tested substances there were either no values less than 50% or no values greater than 50%. The results are presented in Table 2.

**Figure 1:**
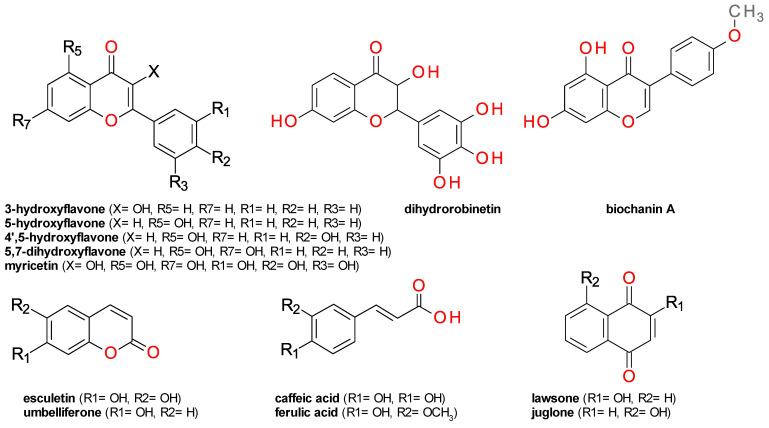
Structure of compounds tested as ColA inhibitors.

Myricetin, and its 3-O-α-L-rhamnopyranoside derivative, myricitrin, have a similar low inhibitory effect on ColA, indicating that the hydroxyl group in position 3 of the flavone scaffold is not essential (Figure 1). The number and position of hydroxyl groups on the flavone scaffold seems to be essential for ColA inhibition. The compounds 3-hydroxyflavone and its 5-hydroxy isomer, primuletin, have a low impact on the enzyme activity, while 4′,5-dihydroxyflavone potently inhibits its activity, indicating the importance of the hydroxyl groups on the phenyl fragment. This is further supported by the low inhibitory effects of 3,6-dihydroxyflavone and 5,7-dihydroxyflavone (chrysin). The relative position of the phenyl moiety is also important, as demonstrated by the potent inhibitory effect of the isoflavone biochanin A and of the 2′-hydroxychalcone.

Caffeic acid; its methoxy derivative, ferulic acid; and its lactone derivative formed by intramolecular cyclization, esculetin, had low effects (Figure 1). All tested coumarins presented low inhibitory effects on ColA, probably because there are no hydroxyls near the ketone group.

Several naphthoquinones and anthraquinones were tested, revealing juglone and rhein as potent inhibitors of ColA. The significant differences between the effect of juglone (Figure S1); 5-hydroxy-1,4-naphthalenedione; and its 2-hydroxy isomer, lawsone, indicate the hydroxyl substitution might influence the inhibitory effect. Juglone, but not lawsone, contains a β-keto enol moiety that is very similar to that of curcumin, a very potent ColA inhibitor (Figure 1). Rhein is an anthraquinone derivative that also contains this structural element, suggesting its importance for enzymatic inhibition.

Capsaicin and piperine have a ketone group. However, this moiety is included in an amidic bond and has no hydroxyl groups in the proximity. Palmatine chloride (Figure S1) is a protoberberine alkaloid with no ketone group or other obvious chemical groups that could explain the inhibition of ColA. We performed a docking study to better understand the inhibition mechanism for these compounds.

A series of amino acids and their derivatives were tested based on their structural analogy with the enzyme’s substrate. Chemically, the compounds were derivatives of alanine (*L*-alanine; N-(3-chlorophenylsulfonyl)-*DL*-alanine), proline (1-{[3-(trifluoromethyl)phenyl]sulfonyl}-2-pyrrolidinecarboxylic acid), captopril, sulbactam, tazobactam), arginine (*L*-arginine, benzoyl-*L*-arginine ethyl ester hydrochloride, nalpha-*p*-toluenesulfonyl-*L*-arginine), and citrulline (*L*-citrulline, Fmoc-*L*-citrulline), cysteine (cysteine, acetylcysteine, and cysteamine hydrochloride). With the exception of citrulline, no other compound of this group had a significant inhibitory effect on ColA activity.

### 3.2. Molecular Docking Simulation

A molecular docking study was performed for 32 compounds to verify the potential correlation between experimentally determined activity values and predicted binding energies. Furthermore, predicted binding poses were analyzed to investigate the potential molecular interactions leading to ColA inhibition. The predicted binding energies varied from −10.025 to −5.429 kcal/mol, with an average value of −8.136 ± 1.060 kcal/mol. Moreover, ligand efficiencies ranged between 0.2788 and 0.6017 (0.4570 ± 0.0842).

Docking results for screened compounds with experimentally determined IC50 values are shown in Table 3.

**Figure 2:**
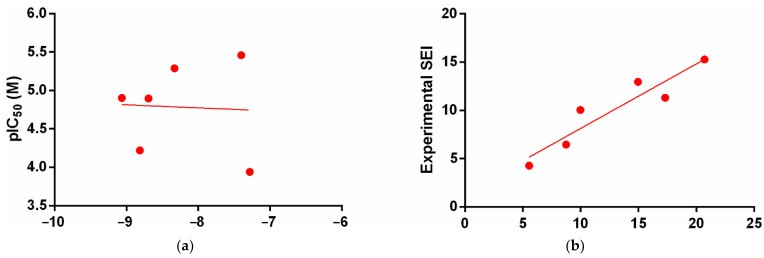
Correlation diagrams between experimental and predicted data. (**a**)—correlation between pIC_50_ values and predicted binding energy; (**b**)—correlation between experimental and predicted SEI.

No correlation could be established between experimentally determined IC50 values and predicted binding energies (R^2^ = 0.0025, *p* > 0.05). Conversely, a strong correlation was observed between experimental and predicted surface-binding efficiency index values (SEI), highlighting the reliability of the docking protocol (R^2^ = 0.8757, *p* = 0.0061, Figure 2). SEI is calculated as the negative logarithmic value of experimental/predicted activity value (pIC50 or pKd) divided to polar surface area/100 Å.

**Figure 3:**
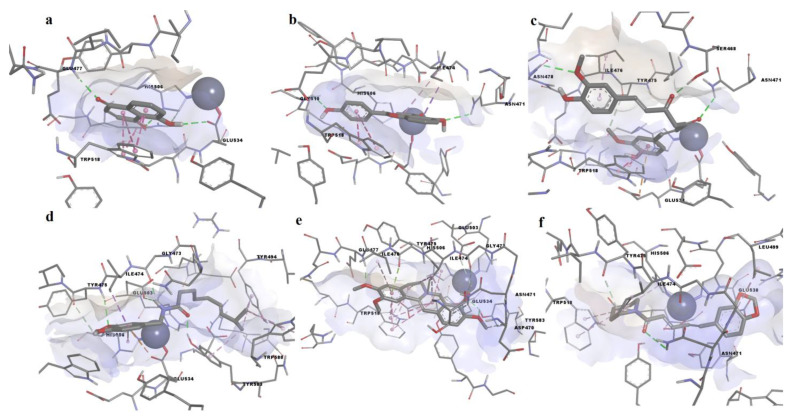
Predicted conformations of the ColA–ligand complexes. (**a**)—juglone; (**b**)—4′,5-dohydroxyflavone; (**c**)—curcumin; (**d**)—capsaicin; (**e**)—palmatine; (**f**)—piperine.

We chose to discuss the binding poses of compounds that exhibited the highest biologic activities in the enzymatic assay in more detail. Among these compounds, curcumin showed the highest predicted binding affinity (−9.185 kcal/mol), followed by 4′,5-dihiydroxyflavone (−9.099 kcal/mol), piperine (−8.404 kcal/mol), palmatine (−8.329 kcal/mol), capsaicin (−8.012 kcal/mol), and juglone (−7.404 kca;/mol). Furthermore, juglone showed the highest ligand efficiency (0.5688). The predicted binding poses of the aforementioned ligands in the ColA active site are shown in Figure 3.

**Figure 4:**
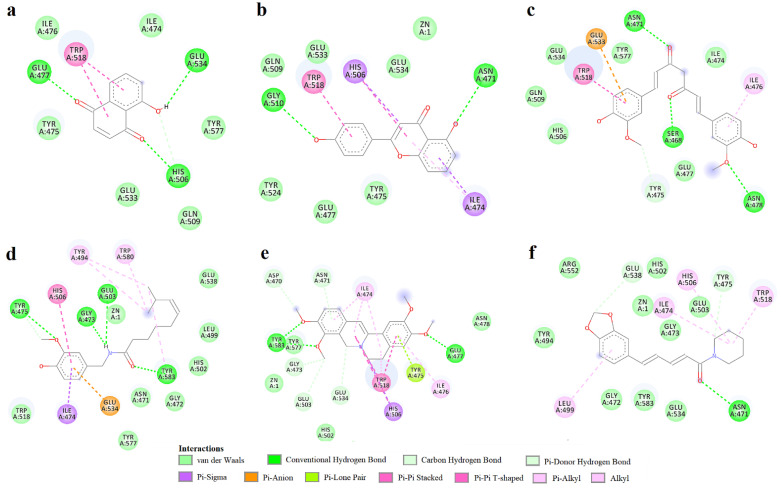
Two-dimensional interaction diagrams for predicted ColA-ligand complexes. (**a**)—juglone; (**b**)—4′,5-dihydroxyflavone; (**c**)—curcumin; (**d**)—capsaicin; (**e**)—palmatine; (**f**)—piperine.

Juglone (Figure 4a) interacted with the binding site by participating in hydrogen bonding with Ca^2+^-binding Glu477 and Zn^2+^-binding residues Gly534 and His506. Moreover, juglone formed pi–pi stacked interactions with Trp518 but did not make any contact with the catalytic zinc. 4′,5-dihydroxyflavone formed hydrogen bonds with Asn471 and Gly510 and pi–sigma and pi–pi T-shaped interactions with the metal-binding His506, while other pi interactions were formed between the aromatic rings and other residues (Figure 4b). Curcumin bound to the active site through hydrogen bonds between the two ketones and Ser468 and Asn471, while forming another hydrogen bond with Asn478 (Figure 4c). Moreover, a pi–anion interaction was formed between a phenyl ring and Glu533. Capsaicin formed 3 hydrogen bonds through the amide moiety, including the polar interaction with the metal-binding Glu503 (Figure 4d). Furthermore, capsaicin formed non-polar interactions with metal-binding His506 (pi–pi T-shaped) and the catalytic zinc (van der Waals forces). Palmatine formed a hydrogen bond with the Ca^2+^-binding Glu477, two other hydrogen bonds with Tyr583 and pi–anion and carbon–hydrogen bonds with the metal-binding Glu503 and Glu534 (Figure 4e). Palmatine also formed pi–sigma interactions with His506 and van der Waals interactions with zinc. Piperine formed a conventional hydrogen bond with Asn471 and two carbon–hydrogen bonds with Glu538 and Tyr475, while being in close contact with zinc (Figure 4f). Moreover, alkyl and pi–alkyl interactions were formed with other relevant residues, such as His506 and Trp518. Interestingly, 4′,5-dihydroxyflavone, curcumin, and piperine bound to the binding pocket by forming hydrogen bonds with Asn471.

Although palmatine and berberine share highly similar structures, berberine adopted a different orientation in the active site, forming no hydrogen bonds with Tyr583. This bond could explain the major difference of effects on ColA between the two alkaloids. The docking study offered additional structure–activity relationships. Unlike 4′,5-dihydroxiflavone, the mono-hydroxy derivatives 3-hydroxyflavone and primuletin were not involved in hydrogen bonding with Gly510, highlighting the importance of this bond. Myricetin, and its rhamnopyranoside derivative myricitrin, could not adopt conformations in the biding pocket that would resemble the binding mode of 4′,5-dihydroxyflavone, possibly due to the additional hydroxyl groups present in their molecules.

## 4. Discussion

The screening revealed the inhibitory effects of 10 compounds with IC50 under 100 μM. Three compounds belong to the class of flavones: dihydrorobinetin, 4′,5-dihydroxyflavone, and biochanin A, all with IC50 values below 12.5 μM. A previous study describes the inhibitory effect of 14 flavone derivatives on collagenase from *Clostridium histolyticum,* revealing quercetin as the best inhibitor with an IC50 value of 286 μM. Myricetin, a compound also tested in our study, presented a similar effect: a 30% inhibition at 100 μM. The authors argue that the 3-hydroxy substituted compounds have a better inhibitory effect [33], but our data seem to contradict this hypothesis—first, because the 3-O-α-*L*-rhamnopyranoside derivative of myricetin, myricitrin, produced similar inhibitory effects, and second because 3-hydroxyflavone and its isomer 5-hydroxyflavone (primuletin) had comparable effects. Our assay results point out the importance of the presence of at least one hydroxyl or methoxy group on the phenyl fragment coupled with the presence of a hydroxyl group either in position 3 or 5. In the docking study, 4′,5-dihydroxyflavone formed hydrogen bonds with Asn471 and Gly510 using its two hydroxyl groups.

The development of dihydrorobinetin as antivirulence agent is limited by its poor solubility in water, while in the case of biochanin A, an impediment may be its estrogen-like effect [34,35]. The 4′,5-dihydroxyflavone emerges as the most promising flavone derivative inhibitor of ColA.

Juglone is a natural naphthoquinone derivative found in several species from the walnut family (*Juglandaceae*) [36]. The inhibitory effect of juglone on ColA coupled with its low impact on the bacterial growth of various bacteria, such as *Staphylococcus aureus*, *Enterococcus faecalis*, and *Staphylococcus epidermidis*, indicating a potentially selective antivirulence profile [37]. The study of juglone toxicity on normal fibrobast cells demonstrated no significant effect at 1 μM concentration [38]. This dose is close to the calculated IC50 on ColA suggesting a low risk of toxicity at small concentrations.

Palmatine chloride is a natural compound reported to have wide spectrum of pharmacological effects, including anticancer, anti-inflammatory, neuroprotection, and antibacterial [39]. A screening study focused on finding inhibitors for the neuraminidase protein of *Clostridium perfringens* identified the potent effect of palmatine [40]. The newly discovered ColA inhibition indicates palmatine chloride as a possible future antivirulence agent with a dual mechanism. An acute toxicity study on mice showed palmatine to be moderately toxic, with a lethal dose 50 of 1533.68 mg/kg [41].

Piperine presented potent inhibitory effects on ColA, even at small concentration. Our results confirm the previous observation on the improvement of the morphological structure of rats’ tendons after the intragastrical administration of piperine (100 mg/kg) in a model where the rats were intratendineously injected with collagenase [42]. The low solubility of piperine limited the calculation of the IC50 value. The use of a self-emulsifying drug-delivery system proved to improve the solubility and oral bioavailability [43] and could represent a future solution for the development of piperine as antivirulence agent.

Both capsaicin and curcumin had IC50 values lower than 5 µM. Capsaicin is a natural occurring vanilloid that was previously shown to possess antimicrobial and antivirulence activities on Group A streptococci [44], while another study revealed its antifungal and antiparasitic potential [45]. Moreover, curcumin was reported in another study to exert antibiofilm, antiefflux and anticapsule activities on hypervirulent *Klebsiella pneumoniae* [46]. Capsaicin and curcumin share common structural features that could explain their inhibitory activity on ColA, such as the vanillyl functional group and the ketone moieties. A large number of clinical trials demonstrated that the administration of both capsaicin and curcumin is safe at therapeutical doses [47,48].

## 5. Conclusions

Capsaicin, 4′,5-dihydroxyflavone, curcumin, dihydrorobinetin, palmatine chloride, biochanin A, 2′-hydroxychalcone, and juglone were identified as promising collagenase inhibitors. Piperine, 4′,5-dihydroxyflavone and capsaicin had IC50 values in the nanomolar range. Our data indicate these molecules could be used against infections caused by multi-drug-resistant bacterial pathogens which express collagenase A.

## Figures and Tables

**Table 1:** The percentage inhibition on ColA after 30 min of incubation.

Compound	Inhibition (%)				
	1 µM	5 µM	10 µM	50 µM	100 µM
Palmatine chloride	15.5 ± 3.9	35.4 ± 1.8	84.9 ± 1.5	97.9 ± 1.6	98.7 ± 0.4
Piperine	84.5 ± 0.4	82.4 ± 0.4	72.5 ± 2.7	NT *	NT *
2′-Hydroxychalcone	−12.1 ± 0.2	1.2 ± 0.9	6.5 ± 1.3	21.8 ± 1.2	97.3 ± 1.3
3-Hydroxyflavone	−13.4 ± 2.7	−17.0 ± 5.1	17.4 ± 4.5	25.1 ± 6.8	31.0 ± 4.3
Myricetin	−15.4 ± 2.7	−4.5 ± 1.1	−1.0 ± 1.1	13.6 ± 0.6	23.2 ± 2.0
Myricitrin	−37.9 ± 2.3	−17.4 ± 2.4	−20.8 ± 1.0	16.7 ± 3.1	37.2 ± 3.8
Dihydrorobinetin	−16.5 ± 1.6	−29.7 ± 0.5	105.1 ± 0.5	NT *	NT *
Primuletin	−9.3 ± 1.7	−8.8 ± 2.1	−0.7 ± 1.3	8.5 ± 2.1	25.4 ± 1.8
4′,5-Dihydroxyflavone	−48.0 ± 3.6	101.6 ± 0.6	107.5 ± 1.0	101.0 ± 1.9	104.8 ± 2.9
Biochanin A	−24.8 ± 5.3	23.0 ± 2.6	34.1 ± 2.2	99.1 ± 1.0	76.1 ± 0.7
Juglone	13.8 ± 1.5	50.6 ± 0.5	69.2 ± 0.4	80.8 ± 0.7	94.8 ± 0.1
Rhein	2.6 ± 0.6	22.9 ± 2.7	37.4 ± 3.2	77.3 ± 2.2	80.6 ± 1.0
Curcumin	−10.0 ± 3.9	101.8 ± 0.6	102.5 ± 0.8	101.5 ± 0.4	101.8 ± 0.7
Capsaicin	94.8 ± 0.8	100.5 ± 0.8	NT *	NT *	NT *
Citrulline	−4.3 ± 3.5	−14.1 ± 1.4	27.8 ± 3.0	24.3 ± 1.7	7.2 ± 0.5
6-Methoxy-1,2,3,4-tetrahydro-9H-pyrido [3,4-b]indole-1-carboxylic acid	−19.9 ± 2.7	−1.3 ± 2.1	−5.2 ± 0.7	2.8 ± 1.3	19.6 ± 3.9

Values are the mean ± standard deviation (SD) of three replications. (*) NT—not tested due to low solubility.

**Table 2:** The IC50 values calculated after 30 min of incubation.

Compound	IC50 (µM)	95% CI of IC50 (µM)	r2
Palmatine chloride	5.139	0.1076 to 245.4	0.918
Piperine	<1	NC *	NC *
2′-Hydroxychalcone	60.395	NC *	NC *
3-Hydroxyflavone	>100	NC *	NC *
Myricetin	>100	NC *	NC *
Myricitrin	>100	NC *	NC *
Dihydrorobinetin	<10	NC *	NC *
Primuletin	>100	NC *	NC *
4′,5-Dihydroxyflavone	<1	NC *	NC *
Biochanin A	12.474	0.2371 to 167.3	0.939
Juglone	3.473	0.5838 to 20.66	0.984
Rhein	12.710	5.225 to 30.92	0.996
Curcumin	<5	NC *	NC *
Capsaicin	<1	NC *	NC *
Citrulline	>100	NC *	NC *
6-Methoxy-1,2,3,4-tetrahydro-9*H*-pyrido [3,4-b]indole-1-carboxylic acid	>100	NC *	NC *
1,10-Phenanthroline	114.810	NC *	NC *

(*) NC— could not be calculated owing to the results obtained, IC50—half maximal inhibitory concentration, 95% CI—95% confidence interval.

**Table 3:** Predicted binding energy, ligand efficiency and amino acid residue contacts.

Compound	LE	ΔG (kcal/mol)	Residue Contacts	Zn Contact
Dihydrorobinetin	0.4313	−9.489	Asn471, Gly472, Gly473, Ile474, Tyr475, Ile476, Glu477, His502, Glu503, His506, Gln509, Gly510, Trp518, Tyr524, Glu533, Glu534, Tyr577, Tyr583	yes
Myricitrin	0.2788	−9.2	Asp470, Asn471, Gly472, Gly473, Tyr485, Arg487, Ser492, Ile493, Tyr494, Leu499, His502, Glu503, Glu538, Tyr577, Gly578, Ser579, Trp580, Phe582, Tyr583	no
Curcumin	0.3358	−9.185	Asn471, Ile474, Tyr475, Ile476, Glu477, Asn478, His506, Gln509, Trp518, Glu533, Glu534, Tyr577	no
4′,5-dihiydroxyflavone	0.4782	−9.099	Asn471, Ile474, Tyr475, Ile476, Glu477, His506, Gln509, Gly510, Trp518, Tyr524, Glu533, Glu534	yes
Biochanin A	0.4316	−9.064	Asp470, Asn471, Gly473, Ile474, Tyr475, Ile476, Glu477, His506, Gln509, Gly510, Trp518, Tyr524, Glu533, Glu534, Tyr577, Tyr583	yes
5-hydroxyflavone (primuletin)	0.5011	−9.02	Asn471, Gly473, Ile474, Tyr475, Ile476, Glu477, Glu503, His506, Gln509, Gly510, Trp518, Tyr524, Glu533	yes
Myricetin	0.3847	−8.847	Asn471, Gly473, Ile474, Tyr475, Ile476, Glu477, Glu503, His506, Gln509, Trp518, Tyr524, Glu533, Glu534, Tyr577, Tyr583	yes
2′-hydroxychalcone	0.5184	−8.813	Ile474, Tyr475, Ile476, Glu477, His506, Gln509, Gly510, Trp518, Tyr524, Glu533	no
Rhein	0.4139	−8.692	Ile474, Tyr475, Ile476, Glu477, Asn478, His506, Trp518, Glu534, Tyr577	no
3-hydroxyflavone	0.468	−8.424	Asn471, Gly473, Ile474, Tyr475, Ile476, Glu477, Glu503, His506, Gln509, Gly510, Trp518, Glu533, Glu534, Tyr577	no
Piperine	0.4079	−8.404	Asn471, Gly472, Gly473, Ile474, Tyr475, Ile476, Tyr494, Leu499, His502, Glu503, His506, Trp518, Glu534, Glu538, Arg552, Tyr577, Trp580, Tyr583	yes
Palmatine	0.3223	−8.329	Asp470, Asn471, Gly473, Ile 474, Tyr475, Ile476, Glu477, Asn478, His506, Met517, Trp518, Gln520, Glu534, Tyr577, Tyr583	yes
Capsaicin	0.3514	−8.012	Asn471, Gly472, Gly473, Ile474, Tyr475, Tyr494, Leu499, His502, Glu503, His506, Trp518, Glu534, Glu538, Tyr577, Trp580, Tyr583, Asn584	yes
6-Methoxy-1,2,3,4-tetrahydro-9*H*-pyrido [3,4-b]indole-1-carboxylic acid	0.4364	−7.855	Asn471, Ile474, Tyr475, Ile476, Glu477, His506, Gln509, Gly510, Trp518, Tyr524, Glu533, Glu534, Tyr577, Tyr583	no
Juglone	0.5688	−7.402	Ile474, Tyr475, Ile476, Glu477, His506, Gln509, Gly510, Trp518, Glu533, Glu534, Tyr577	no
1,10-Phenanthroline	0.5203	−7.284	Asn471, Gly472, Gly473, Tyr494, Leu499, His502, Glu503, Glu538, Trp580, Tyr583	no
Citrulline	0.4984	−5.981	Ile474, Tyr475, Ile476, Glu477, His506, Gln509, Gly510, Val514, Trp518, Tyr524, Glu533, Glu534, Tyr577	no

LE—ligand efficiency.

## Data Availability

Not applicable.

## Supplementary Materials

The following supporting information can be downloaded at: https://www.mdpi.com/article/10.3390/life12122114/s1, Figure S1: The inhibitory effect of two compounds on ColA after 30 min. (a) juglone; (b) palmatine chloride; Table S1: The percentage inhibition on ColA after 30 min of incubation at 100 µM.
