# Supplementary material for: Discovery of New Microbial Collagenase Inhibitors
Source: Life (Basel). 2022 Dec 15;12(12):2114. doi: 10.3390/life12122114 (PMC9781087; doi:10.3390/life12122114)
Supplement: Supplementary file 1 [file life-12-02114-s001.zip › life-2047989-supplementary.pdf]

Supplementary Materials

Discovery of New Microbial Collagenase Inhibitors

Georgiana Nitulescu [1], Dragos Paul Mihai [1,*], Anca Zanfirescu [1], Miruna Silvia Stan [2,3], Daniela Gradinaru [1] and George Mihai Nitulescu [1]

[1] Faculty of Pharmacy, "Carol Davila" University of Medicine and Pharmacy, Traian Vuia 6, Bucharest 020956, Romania
[2] Department of Biochemistry and Molecular Biology, Faculty of Biology, University of Bucharest, 91–95 Spl. Independentei, Bucharest 050095, Romania
[3] Research Institute of the University of Bucharest (ICUB), University of Bucharest, Bucharest 050657, Romania
* Correspondence: dragos_mihai@umfcd.ro

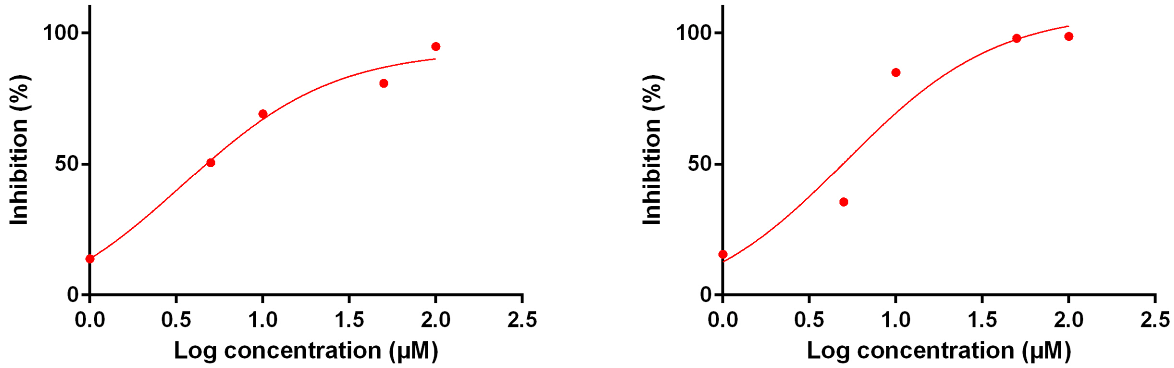

(**a**) (**b**)

Figure S1. The inhibitory effect of two compounds on ColA after 30 min. (**a**) juglone; (**b**) palmatine chloride.

Table S1. The percentage inhibition on ColA after 30 min of incubation at 100 µM.

Compound	Inhibition (%)
1,2,3,4-tetrahydro-beta-carboline-1-carboxylic acid	1.5
1,2-diaminoanthraquinone	4.3
1,4-dihydroxyanthraquinone	3.2
1-{[3-(trifluoromethyl)phenyl]sulfonyl}-2-pyrrolidinecarboxylic acid	0.7
2'-hydroxychalcone	87.8
3,6-dihydroxyflavone	2.7
3-hydroxycinnamic acid	0.8
3-hydroxyflavone	27.6
3-phenyl-1H-2-benzopyran-1-one	0.2
4',5-dihydroxyflavone	15
4,6-dihydroxy-2-mercaptopyrimidine	-7.2
4'-methoxyflavonol	3.7
4-methylphenylsulfonylurea	19.6
6-hydroxycoumarin	12.5
6-methoxy-1,2,3,4-tetrahydro-9H-pyrido[3,4-b]indole-1-carboxylic acid	18.8
6-methoxyflavonol	10.7
acamprosate calcium	17.2

acetohexamide	16.6
acetylcysteine	3.4
aloin	4.4
apigenin	2.7
argatroban	-12.3
artemisinin	5.6
bendroflumethiazide	21.6
benfotiamine	-20.4
benzoyl-L-arginine ethyl ester hydrochloride	-6.6
berberine	-2.5
biochanin A	57.9
caffeic acid	-3.8
cantharidin	26.8
capsaicin	87.9 *
captopril	5.2
chlorogenic acid	4.8
chrysin	-3.4
chrysophanol	-11.1
citrulline	6.8
curcumin	102.1
cysteamine hydrochloride	2.8
cysteine	1.7
daidzein	5.8
daidzein dimethyl ether	15.7
dihydrorobinetin	96.9 *
epicatechin	3.6
esculetin	11.8
etidronic acid	23
ferulic acid	0.5
Fmoc-L-citrulline	20.8
gallic acid	-21.4
genistein	-1.1
isorhamnetin	19.9
isoxicam	-10.1
juglone	89.9
L-alanine	3.1
L-arginine	-12.7
lawsone	-6.1
meso-2,3-dimercaptosuccinic acid	25.6
myricetin	27.6
myricitrin	26.1
N-(3-chlorophenylsulfonyl)-DL-alanine	-19.2
nalpha-p-toluenesulfonyl-L-arginine	24.8
nimesulide	-2.2
palmatine	103.4
piperine	81.1 *
plumbagin	-7.7
podophyllotoxin	-9.7

potassium clavulanate	-8.1
primuletin	33.1
rhein	78.5
S-(acetamidomethyl)-L-cysteine	-15.2
shikimic acid	-0.2
sodium 2- mercaptoethanesulfonate	20.6
sulbactam	-10.7
tazobactam	18.7
tetrahydronorharman	21.3
trihydroxyethylrutin	1.3
umbelliferone	-10.2
usnic acid	3.9

(*) the compounds precipitated due to their low solubility
